# Injecting hope: the potential of intratumoral immunotherapy for locally advanced and metastatic cancer

**DOI:** 10.3389/fimmu.2024.1479483

**Published:** 2025-01-09

**Authors:** Marketa Skalickova, Katerina Hadrava Vanova, Ondrej Uher, Jindriska Leischner Fialova, Katerina Petrlakova, Michal Masarik, Zdeněk Kejík, Pavel Martasek, Karel Pacak, Milan Jakubek

**Affiliations:** ^1^ BIOCEV, First Faculty of Medicine, Charles University, Vestec, Czechia; ^2^ Department of Paediatrics and Inherited Metabolic Disorders, First Faculty of Medicine, Charles University and General University Hospital, Prague, Czechia; ^3^ Section on Medical Neuroendocrinology, Eunice Kennedy Shriver National Institute of Child Health and Human Development, National Institutes of Health, Bethesda, MD, United States; ^4^ Department of Pathological Physiology, Faculty of Medicine, Masaryk University, Brno, Czechia; ^5^ Department of Physiology, Faculty of Medicine, Masaryk University, Brno, Czechia

**Keywords:** cancer, immunotherapy, intratumoral, combination therapy, advanced and metastatic cancer

## Abstract

Despite enormous progress, advanced cancers are still one of the most serious medical problems in current society. Although various agents and therapeutic strategies with anticancer activity are known and used, they often fail to achieve satisfactory long-term patient outcomes and survival. Recently, immunotherapy has shown success in patients by harnessing important interactions between the immune system and cancer. However, many of these therapies lead to frequent side effects when administered systemically, prompting treatment modifications or discontinuation or, in severe cases, fatalities. New therapeutic approaches like intratumoral immunotherapy, characterized by reduced side effects, cost, and systemic toxicity, offer promising prospects for future applications in clinical oncology. In the context of locally advanced or metastatic cancer, combining diverse immunotherapeutic and other treatment strategies targeting multiple cancer hallmarks appears crucial. Such combination therapies hold promise for improving patient outcomes and survival and for promoting a sustained systemic response. This review aims to provide a current overview of immunotherapeutic approaches, specifically focusing on the intratumoral administration of drugs in patients with locally advanced and metastatic cancers. It also explores the integration of intratumoral administration with other modalities to maximize therapeutic response. Additionally, the review summarizes recent advances in intratumoral immunotherapy and discusses novel therapeutic approaches, outlining future directions in the field.

## 1 Introduction

Today, cancer ranks as the second leading cause of mortality worldwide (1) with predicted incidence expected to reach 28.4 million cases by 2040 (2). Locally advanced tumors (those that have significantly progressed in size or are often inoperable due to locoregional spread) and metastatic tumors (those that have spread to distant parts of the body) (3) are the primary causes of cancer-related death (4). According to the Surveillance, Epidemiology, and End Results database (2014–2020), poor 5-year survival rates are documented for metastatic and regional diseases, such as 99.6% for localized, 86.7% for regional, and 31.9% for distant female breast cancer (5).

One of the main challenges in cancer treatment is finding effective modalities to combat both primary and distant metastatic tumors, as well as addressing post-treatment minimal residual disease (MRD), which includes small cancer cell clusters, micrometastasis, or even single cancer cells. While advances in liquid biopsy for detecting MRD are promising in hematological malignancies, they remain challenging for many solid tumors (6, 7). The unpredictable development of metastasis, proximity to vital organs or vessels, as well as large tumor size often complicate the treatment of advanced cancers (3). Generally, the size of a lesion, the number of affected lymph nodes, and the extent of metastasis correlate with a worse prognosis, also known as the tumor, node, and metastasis (TNM) staging system (8).

Current treatment options for locally advanced and metastatic cancer include systemic and local therapies or their combinations, depending on the type, localization, and stage of cancer progression (9–14). According to the National Cancer Institute, Bethesda, Maryland, systemic cancer treatment nonspecifically travels through the bloodstream and affects both cancerous and healthy cells (15), while local therapies target cancer cells with reduced toxicity to nearby and distant healthy cells (16). Systemic treatment options include chemotherapy, immunotherapy, hormonal, and specifically targeted therapies (17), such as epigenetic drugs, tyrosine kinase inhibitors, or anti-angiogenesis drugs (18). Local treatments comprise surgery, radiotherapy (9, 13, 19–21), photodynamic (22), and ablation therapies (9, 13, 21).

Although side effects are observed with both treatment types (23, 24), systemic therapies are often the leading cause of bone marrow suppression, gastrointestinal dysfunction, endocrine abnormalities (25), and immune-related adverse events (26). Given these challenges, there is an urgent need for new treatment strategies and combinations that lower side effects, achieve synergistic or additive activity, and increase efficacy for treating locally advanced and metastatic cancer (27). This review provides an up-to-date overview of current immunotherapeutic and other treatment approaches, focusing on intratumoral administration for the treatment of locally advanced and metastatic cancer. We emphasize combination therapy, where intratumoral administration is paired with other modalities, to improve the overall therapeutic response. By examining the latest advancements, particularly in immunotherapy, we also discuss novel, promising therapeutic approaches for the near future.

## 2 Cancer immunotherapy

The involvement of the immune system in tumor development was first proposed in 1909. This idea was further studied about 50 years later by F. M. Burnet and L. Thomas. They hypothesized that tumor neoantigens could trigger T cell immune response to eliminate cancer cells (28). This hypothesis was validated in the 1990s by numerous experiments, extending to the interplay between innate and adaptive immunity necessary for efficient tumor eradication (29–31) and the generation of immunological memory to prevent disease recurrence (32).

Cancer immunotherapy is a promising approach that enhances tumor immunogenicity and stimulates the immune response against cancer cells (33, 34). For decades, researchers have explored ways to stimulate or inhibit immune response to fight cancer. Currently, several types of immunotherapies are recognized, including cancer vaccines (e.g., dendritic cell, peptide/protein, gene (35), viral (36), oncolytic viral (37), or repurposed viral vaccines) (35), monoclonal antibodies and checkpoint inhibitors (ICIs) (38), adoptive cell therapies (39, 40), and immunomodulators (e.g., cytokines, pattern recognition receptor (PRR) and stimulator of interferon genes (STING) agonists, and vaccine adjuvants) (41). Based on their modulation of the immune system, they can be categorized as either active or passive, although many immunotherapies exhibit overlaps. Active immunotherapy is based on a patient’s immunization with pathogen vaccines, tumor cells or their parts, ICIs, or cytokines, with a subsequent generation of various immune mediators and cells to destroy a tumor lesion, ultimately through effector T cells. Passive immunotherapy, on the other hand, includes the administration of *ex vivo* stimulated or modified immune cells, such as T cells, natural killer (NK) cells, or chimeric antigen receptor T cell therapy (CAR-T), as well as the administration of specific monoclonal antibodies, without the necessity to initiate the production of own immune mediators and cells to fight cancer (42, 43). Depending on their immunostimulatory activity, monoclonal antibodies can be considered either targeted therapy (44) or immunotherapy (45).

To date, numerous systemic immunotherapies have been approved for the treatment of advanced cancers (National Library of Medicine Database, European Medicine Agency Database). Systemic administration is essential for effectively managing metastatic disease (46), as it is both practically feasible (47) and ensures a broad distribution of therapeutic agents throughout the body (46). These primarily include ICIs and their combinations with chemotherapy, targeted therapy (e.g., vascular endothelial growth factor (VEGF), kinase, and epidermal growth factor receptor (EGFR) inhibitors), or additional ICI molecules. Other treatment options include, for instance, sipuleucel-T (FDA, viral therapy), aldesleukin (FDA, cytokine therapy), lifileucel (FDA, adoptive cell therapy), or enfortumab (FDA/EMA, anti-nektin-4 antibody). Although these therapies have been approved for managing advanced cancers, they are not combined with other treatment modalities.

## 3 Intratumoral immunotherapy

**Figure 1:**
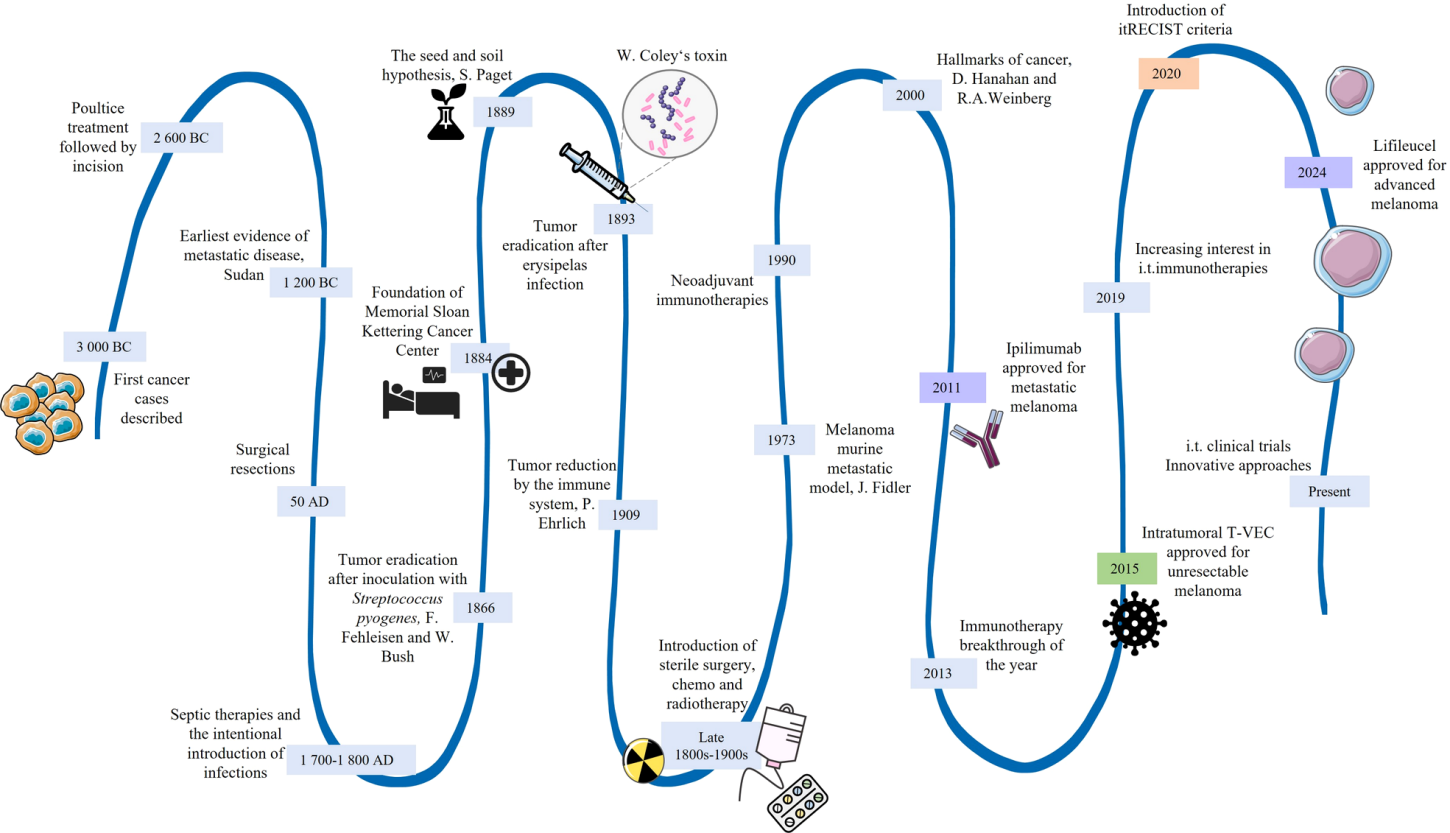
Historical milestones of the intratumoral approach, including the key events for advanced cancers. i.t. intratumoral.

The beginning of intentional local treatments can be dated back to 1700 – 1800 AD (48), almost 5,000 years after the first cases were documented in old papyruses and treated (49). In the case of metastatic disease, the earliest evidence is from 1200 BC (50) (**Figure 1**). As with any of the standard cancer treatments, the introduction of immunotherapy, including the intratumoral approach, was a gradual process developing with new-gained knowledge and ever-changing discoveries in the biological field. Significant advances in cancer research and treatments began to emerge with the foundation of the first hospital for cancer patients, Sloan Kettering Institute (currently Memorial Sloan Kettering Cancer Center), in 1884 (51) (**Figure 1**). For instance, several cases describing tumor eradication after infections were reported by F. Fehleisen, W. Bush (52), and W. Coley (52–54). Although not well understood at that time, Coley’s work illuminated the potential of direct tumor injection to stimulate the immune system to fight both primary tumors and metastases (38). Furthermore, the “seed and soil” hypothesis of organ-specific metastatic dissemination was proposed (55) (**Figure 1**). A few years after the postulation that the immune system could reduce tumor growth (28), sterile procedures (56, 57) were introduced, leaving W. Coley misunderstood and forgotten. Additionally, the first metastatic mouse melanoma model was established (58). With further scientific advances, hallmarks of cancer (59) and the neoadjuvant approach were introduced (60), immunotherapy was named the Breakthrough of 2013 (61), and itRECIST criteria, i.e., recommendations for the assessment of intratumoral immunotherapy clinical trials, was proposed *(in lorange) (* 62) (**Figure 1**). The induction of systemic immune response against distal untreated lesions, better known as abscopal or anesthetic effects, was sometimes reported during better-defined radiotherapy (63, 64). For a long time, only systemic treatment was considered necessary to reach all metastatic lesions. However, to specifically target cancer cells, reduce off-target toxicities, and increase treatment efficacy, a focus has shifted again towards local, particularly intratumoral immunotherapies (47, 65). The first approved systemic monoclonal antibody and cell-based therapy for advanced cancer were ipilimumab (FDA/EMA) (66) and lifileucel (FDA) (67), respectively *(in purple)*. However, to this day, the only FDA/EMA-approved intratumoral immunotherapy is Talimogene Laherparepvec (T-VEC) (68), which is used for the treatment of unresectable melanoma lesions *(in green)* (**Figure 1**). Herpes virus G47∆ has been approved particularly by the Japan Ministry of Health, Labour and Welfare for the intratumoral treatment of malignant glioma (69) and adenovirus H101 in China for advanced nasopharyngeal cancer (70).

Intratumoral immunotherapy is based on the principle of *in situ* immunization, where the immune response is primed directly within the tumor microenvironment. This can involve the stimulation of pre-existing anti-tumor immunity or the initiation of the new, often tumor-specific immune responses (65, 71, 72). The specific mechanisms activated depend on several factors, including the type of immunotherapeutic agent used, its biological activity, timing, and combination with other therapies (47, 65, 71). One of the common mechanisms is the activation of dendritic cells within the tumor through intratumoral injection of immunotherapeutic agents. These activated dendritic cells present tumor antigens to T cells, initiating a robust anti-tumor immune response (73, 74). Another mechanism involves the injection of agents such as cytokines or cytokine-inducing compounds into the tumor, which modulate the local immune environment and promote the recruitment and activation of immune cells (75, 76). Additionally, intratumoral administration of checkpoint inhibitors can block inhibitory signals within the tumor microenvironment, enhancing the activity of T cells against cancer cells (77, 78). Oncolytic viruses, which selectively infect and kill tumor cells, also play a role by releasing tumor antigens and stimulating a broader immune response (79). Approved or investigational systemic immunotherapies can be intended for local administration if accessible tumor lesions (i.e., primary tumors or metastases) are present for direct injection via skin, surgery, or any of the endoscopic procedures, and pending their potential future approval for use in local treatment settings (71).

Intratumoral administration exerts several advantages over the traditional systemic approach: (a) It significantly reduces the exposure of immunotherapeutic drugs to healthy tissues, thereby lowering the likelihood of side effects (47, 65, 72, 80). (b) It enables combinations of drugs that may be excessively toxic if administered systemically (65, 80). (c) It is easier to achieve high intratumoral bioavailability (47, 65, 72). (d) It allows for lower doses, reducing costs while maintaining therapeutic efficacy (65, 80). However, intratumoral immunotherapy also has its disadvantages. Not all tumors are accessible for direct injection, limiting the applicability of this approach to certain cancer types and locations (81, 82). Local treatments can cause reactions at the injection site, including undesired pain, swelling, and inflammation (83–85). The variability within the tumor microenvironment can also affect the uniformity and effectiveness of the treatment (86). Moreover, the administration of intratumoral immunotherapies requires precise delivery techniques, such as image-guided injections, which can complicate the process (47, 81, 87). Contrary to systemic therapies, intratumorally administered agents may not reach undetected lesions (88–90). Finally, while strong local immune activation is an advantage, it may not always result in a sufficiently robust systemic response to control metastatic disease (91). This risk of insufficient systemic response remains a challenge in effectively managing cancer with intratumoral immunotherapy.

## 4 Rationale for combination therapy in the treatment of advanced cancer

In the last two decades, the introduction of concepts such as the “Hallmarks of Cancer” and the “Cancer-Immunity Cycle” has profoundly influenced the understanding and treatment of cancer. Despite the natural ability of the innate and adaptive immune system to recognize and eliminate cancer, malignant/metastatic cells have developed numerous evading mechanisms. These hallmarks were described by Hanahan and Weinberg to conceptualize cancer as a complex tissue of cells communicating with each other. Currently, fourteen hallmarks of cancer have been introduced, including (1): evading growth suppressors (2), non-mutational epigenetic reprogramming (3), genome instability and mutations (4), avoiding immune destruction (5), inducing or accessing vasculature (6), resisting cell death (7), activating invasion and metastasis (8), tumor-promoting inflammation (9), enabling replicative immortality (10), polymorphic microbiomes (11), senescent cells (12), unlocking phenotypic plasticity (13), deregulating cellular metabolism, and (14) sustaining proliferative signaling (**Table 1**) (59, 146, 147).

**Table 1:** Therapeutic approaches inducing ICD and targeting the Hallmarks of Cancer.

Therapies Triggering Immunogenic Cell Death	
Agonists	BO-112 (TLR3 agonist) (92), RIG-I-like helicases (93), imiquimod (TLR7 agonist) (94), BCG (TLR9 agonist) (95)
Cancer vaccines	Talimogene laherparepvec (96), adenovirus (97), reovirus (98), Newcastle disease virus (99)
Ablative therapies	cryotherapy (100), hyperthermic therapy (101), photodynamic therapy (102), irreversible electroporation (103)
Chemotherapy	bleomycin, doxorubicin (104), pemetrexed (105), oxaliplatin (106), mitoxantrone (107), paclitaxel (108, 109)
Targeted therapy	proteasome inhibitor bortezomib (110), anti-EGFR antibody cetuximab (111), CDK inhibitor dinaciclib (112), ALK inhibitor crizotinib, TKI inhibitor foretinib (113), proteasome inhibitor carfilzomib (114)
Radiotherapy	(115)
Hormone therapy	progesterone inhibitor mifepristone (116)
Therapies Targeting Hallmarks of Cancer	
Evading growth suppressors	PARP inhibitors (117), CDK inhibitors (118, 119)
Non-mutational epigenetic reprogramming	DNA methyltransferase inhibitors, histone deacetylase inhibitors (120, 121)
Genome instability and mutations	PARP inhibitors (122), topoisomerase inhibitors (123), inhibitors of microsatellite instability (124), checkpoint kinase inhibitors (125), CRISPR/Cas9 (126), checkpoint inhibitors (127)
Avoiding immune destruction	cell adoptive therapies, immunomodulators, monoclonal antibodies (128), peptide cancer vaccines (129), checkpoint inhibitors (128)
Inducing or accessing vasculature	anti-VEGF/VEGFR, TKIs, PI3K inhibitors, AKT inhibitors, ERK inhibitors, mTOR inhibitors (130)
Resisting cell death	Bcl-2 and Bcl-xl inhibitors, Mcl1 inhibitors, IAP Inhibitors, SMAC mimetics (131), BH3 mimetics (132)
Activating invasion and metastases	migrastatics (133), MMP inhibitors (134), integrin inhibitors (135)
Tumor promoting inflammation	cytokine/cytokine receptor inhibitors (e.g., anti-IL6, anti-TGFβ anti-TGFRβ, anti-TNF/TNFR), COX2 inhibitors, anti-infection agents (136)
Enabling replicative immortality	telomerase inhibitors, human telomerase reverse transcriptase inhibitors (137), cytotoxic gene therapy (138)
Polymorphic microbiomes	probiotics, prebiotics, fecal microbiota transplantation (139)
Senescent cells	senolytics and senomorphics (140)
Unlocking phenotypic plasticity	TGFβ inhibitors, Src inhibitors, VEGF inhibitors, MMP inhibitors (141, 142), epigenetic drugs (e.g., HDAC inhibitors) (143)
Deregulating cellular mechanism	inhibitors of glucose, glutamine, fatty acid, or nucleotide metabolism (144)
Self-sufficiency in growth signals	PDGF inhibitors, EGFR inhibitors (145)

PARP, poly adenosine diphosphate-ribose polymerase; CDK, cyclin-dependent kinase; DNA, deoxyribonucleic acid; CRISPR/cas9, clustered regularly interspaced short palindromic repeats/associated protein 9; VEGF, vascular endothelial growth factor; VEGFR, vascular endothelial growth factor receptor; PI3K, phosphoinositide 3-kinase; AKT, protein kinase B; ERK, extracellular signal-regulated kinase; mTOR, mammalian target of rapamycin; Bcl-2 B-cell lymphoma 2, Bcl-xl B-cell extra large lymphoma, Mcl1 myeloid cell leukemia 1; IAP, inhibitor of apoptosis; SMAC, second mitochondria-derived activator of caspase; BH3, Bcl-2 homology domain 3; MMP, matrix metalloproteinase; TGFβ, transforming growth factor beta; TGFRβ, transforming growth factor receptor beta; TNF, tumor necrosis factor; TNFR, tumor necrosis factor receptor; TLR, toll-like receptor; IL, interleukin; COX2, cyclooxygenase-2; HDAC, histone deacetylase; PDGF, platelet-derived growth factor; EGFR, epidermal growth factor receptor; TKI, tyrosine kinase inhibitor.

**Figure 2:**
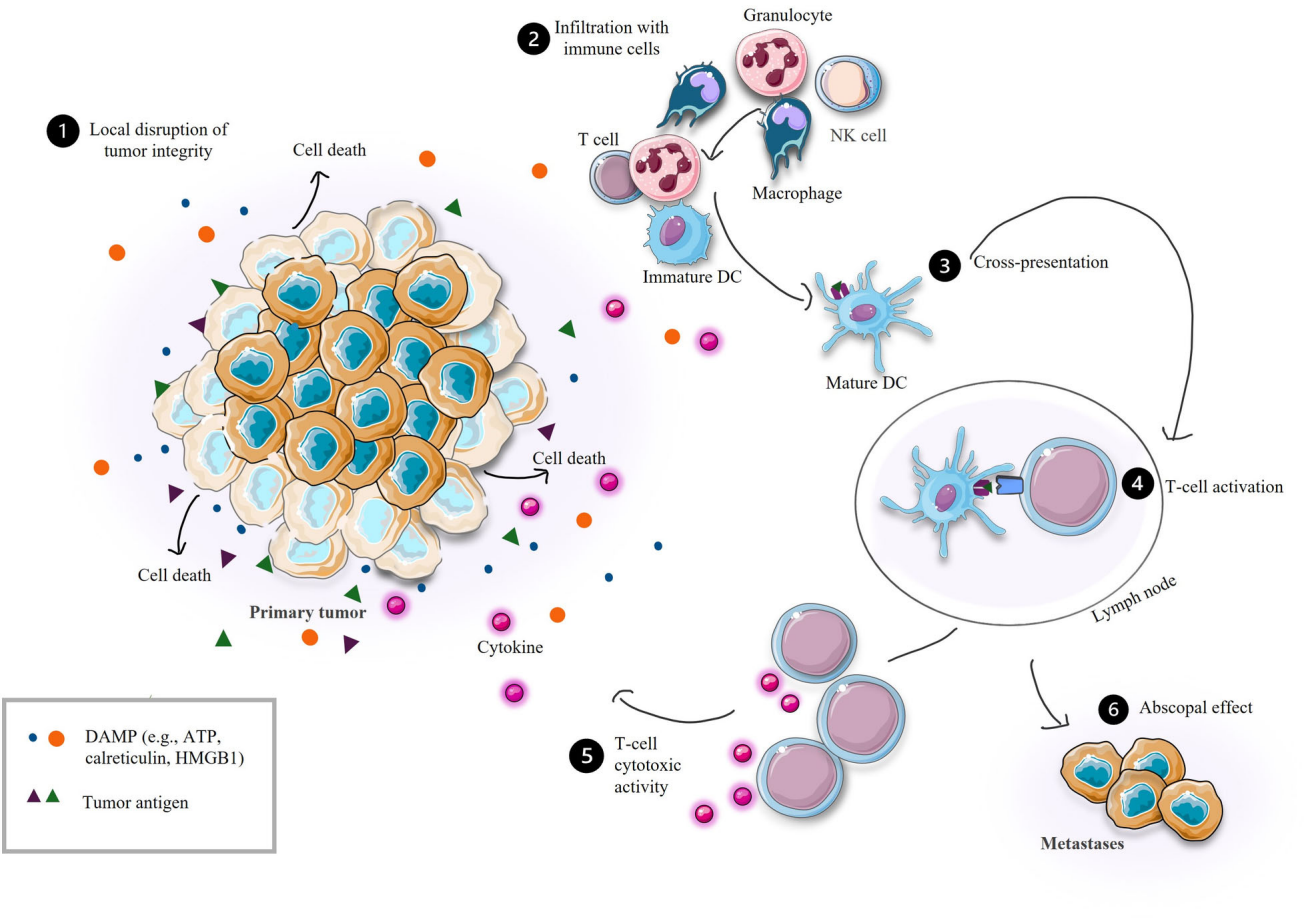
Efficient anti-tumor immune response. DC, dendritic cell; NK, natural killer; DAMP, damage-associated molecular pattern; ATP, adenosine triphosphate; HMGB1, high mobility group box 1 protein.

In 2013, Chen and Mellman introduced a novel concept, the “Cancer-Immunity Cycle”, which describes crucial steps for an efficient anti-tumor immune response (148). The critical step of the cycle is the induction of local disruption of tumor cells, followed by the release of specific tumor motives such as tumor antigens, damage-associated molecular patterns (DAMPs), pathogen-associated molecular patterns (PAMPs), and various pro-inflammatory cytokines (148, 149). These released tumor motives consequently attract the cells of innate immunity (e.g., macrophages or dendritic cells (DCs)) that start to engulf and present tumor antigens with major histocompatibility complex (MHC) molecules. Stimulated antigen-presenting cells (APCs) then migrate to draining lymph nodes where they present tumor antigen-MHC complexes to naïve T cells, activating them in an antigen-specific manner. This leads to the infiltration of the primary tumor by activated adaptive immunity cells, which subsequently destroy tumor cells. This process further triggers the release of tumor motives and pro-inflammatory signals (148). Tumor cells in distant lesions, i.e., metastases, may be eradicated under certain conditions (**Figure 2**) (150, 151).

Taking these concepts in mind, cancer treatment for advanced and metastatic tumors should target two or more “hallmarks of cancer” (152–154) while incorporating at least one approach that stimulates immunogenic cell death (ICD) to enhance efficacy and increase the immunogenicity of cancer cells (155). ICD is a regulated form of cell death characterized by membrane rupture, the release of molecules such as adenosine triphosphate (ATP), high mobility group box 1 protein (HMGB1), annexin 1, heat shock protein (HSP), type I interferon, cytosolic DNA/RNA, tumor antigens, and the translocation of calreticulin to the cell membrane (156, 157). Several types of ICD have been identified, including immunogenic apoptosis, necrosis, and pyroptosis (155), as well as recently proposed ferroptosis, parthanatos, immunogenic entotic or netotic cell death, lysosome- and autophagy-dependent cell death, and alkaliptosis (158). ICD has also been described for immunotherapeutic approaches. Examples of therapies inducing ICD are summarized in **Table 1**. While it may be reasonable to expect ICIs or cell therapies to indirectly induce ICD via immune cell mediators, studies on the release of DAMPs have primarily focused on established ICD inducers without examining the potential of ICIs or cell therapies alone (159, 160).

## 5 Combinations of intratumoral immunotherapies with other treatment modalities

### 5.1 Intratumoral cancer vaccines

Cancer vaccines are a diverse group of therapies that include dendritic cells, peptide/protein, gene, viral, oncolytic viral or repurposed viral vaccines (35) (**Table 2**). They aim to stimulate both the innate and adaptive arms of the immune system (35, 164) and are typically combined with immune adjuvants to enhance the immune response (35, 164). Despite the development of numerous cancer vaccines and their modification, their efficacy is often reduced in advanced and metastatic cancers (165, 166). Therefore, combining them with other therapeutic approaches is essential (167).

**Table 2:** Classification of cancer vaccines.

Type of Vaccine	Dendritic Cell Vaccine	Peptide/Protein Vaccine	Gene Vaccine	Viral Vaccine	Oncolytic Viral Vaccine	Repurposed Viral Vaccine
Description	Dendritic cells loaded with tumor neoantigens (36)	Delivery of tumor neoantigen epitopes (36)	DNA/RNA encoding tumor neoantigens (36)	Viral vectors encoding tumor neoantigen (36)	Oncolytic viral particles (161, 162)	Already approved viral- disease-preventable vaccines (163)

#### 5.1.1 Viral repurposed and viral oncolytic vaccines

A promising approach in cancer vaccines is drug repurposing, which involves identifying new therapeutic uses for existing medications. Existing knowledge of safety profiles, pharmacokinetics, and manufacturing processes can expedite the introduction of new treatments to the market (168). Repurposed vaccines, such as diphtheria, influenza, measles, smallpox, and yellow fever vaccines, have been explored for their potential anticancer properties (169). For example, intratumoral administration of the influenza vaccine in a murine metastatic breast cancer model induced acute inflammation, reduced tumor size, and reversed resistance to systemic ICIs, leading to reduced metastases (170). There is also a clinical trial underway to evaluate the safety of influenza vaccination among breast cancer patients receiving chemotherapy in the neoadjuvant setting (NCT06229392). In phase I of this study (NCT06229392), 2 doses of seasonal flu vaccine will be administered to breast cancer tissue, and the tumor and whole body response will be studied. Similarly, in a bilateral colorectal murine model, intratumoral administration of the yellow fever vaccine combined with systemic administration of anti-programmed cell death protein 1 (anti-PD-1) and anti-CD137 reduced tumor growth. Interestingly, mice that were preimmunized with the same vaccine demonstrated enhanced local and distant antitumor immunity (171), highlighting the potential of widespread vaccine deployment.

In addition to repurposing vaccines, researchers investigated the concurrent intratumoral administration of various oncolytic viruses and bacteria alongside other therapies to amplify the antitumor effects in various preclinical models (172) and clinical studies targeting advanced disease (**Supplementary Table 2**). Numerous oncolytic viruses, such as coxsackievirus (NCT02307149), dengue virus (NCT03990493), and vaccinia virus (NCT05859074), are currently used for the *in situ* immune system activation in clinical trials (173) (**Supplementary Table 1**). However, results from clinical trials using oncolytic viruses alone as cancer vaccines often yielded disappointing outcomes (174). Therefore, combination strategies are preferred to enhance the effectiveness of oncolytic immunotherapies (173). Combining oncolytic virus therapy with ICIs in preclinical models of several cancers has shown significantly prolonged survival compared to untreated mice or those receiving either therapy alone (175–177). Given that ICIs are extensively employed in treating locally advanced and metastatic cancers, most clinical trials focus on combining oncolytic immunotherapies with the systemic administration of ICIs (**Supplementary Table 1**). Furthermore, intratumorally administered oncolytic adenovirus VCN-01 has shown encouraging biological and clinical activity when administered with chemotherapy to patients with pancreatic adenocarcinoma (PDAC) (NCT02045589). VCN-01 was administered by endoscopic ultrasound guidance to the primary lesion. In all treated patients, the injected lesion remained stable or decreased in size. Of the seven evaluable patients, five experienced progression at 4 months, one at 8 months, and one at 31 months after treatment. Progression in all patients was due to the appearance of new lesions or the growth of distant, non-injected metastatic lesions (178). In 2023, the FDA granted Fast Track designation to intravenously administered VCN-01 for treating metastatic PDAC (NCT05673811), following promising results from previous studies combining intravenous VCN-01 with chemotherapy for PDAC treatment (NCT02045602) (179).

Advances in genetic engineering have led to the development of recombinant viruses that attract immune cells to infiltrate the tumor and deliver additional tumor antigens and immunomodulators. Consequently, their activity triggers T cell activation and enhances the anti-tumor immune response. T-VEC, the first FDA/EMA-approved oncolytic virus for intratumoral application, is an example of a genetically engineered herpes virus with an additional gene for human granulocyte-macrophage colony-stimulating factor (GM-CSF), showing encouraging outcomes for several advanced tumors (180, 181). In clinical trial NCT02509507, T-VEC was injected intratumorally in 21-day cycles with intravenous pembrolizumab, and a feasible and tolerable combination was demonstrated to continue further investigation (182). Besides incorporating the gene for GM-CSF into viral vectors in different oncolytic viruses (NCT02562755, NCT05162118, NCT04050436), viruses expressing cytokines such as IL-12 and IL-15 or ICIs are currently tested (NCT06008925, NCT05081492, NCT04370587, NCT04735978, NCT06124001).

In clinical trials, virotherapies are typically administered through either intratumoral or intravenous routes. While intratumoral administration is expected to result in fewer adverse events and limited accumulation of viral particles within metastatic lesions, intravenous administration faces challenges such as restricted penetration of viral particles through dense tumor stroma and an immunosuppressive microenvironment, along with rapid serum degradation by the immune system (183). However, a few studies have reported comparable results between local and systemic administration (184). In the case of repurposed viral vaccines, intratumoral administration may offer an advantage by preventing the rapid elimination of viral particles due to neutralizing antibodies and memory cells (183). The limited number of clinical trials directly comparing the safety and efficacy of intratumoral versus systemic administration highlights an urgent need for more comprehensive studies to fill this critical gap in research.

#### 5.1.2 Peptide/protein and dendritic cell vaccines

Intratumoral peptide/protein and dendritic cell vaccines represent promising strategies for the treatment of advanced cancers (185, 186). They involve the administration of tumor-specific antigens, i.e., neoantigens or peptides and *ex vivo* primed dendritic cells, respectively, to stimulate the adaptive arm of the immune system to combat cancer (36). However, in the advanced disease setting, combining other treatment modalities is essential for effective treatment (186, 187).

Dendritic cancer vaccines are often investigated in combination with systemic checkpoint inhibitors (e.g., NCT03942328, NCT03546361, NCT03707808). Several clinical trials also examined the efficacy of intratumorally delivered DCs with a kinase inhibitor for the treatment of metastatic renal cell carcinoma (NCT02432846, 2014-004510-28). For instance, the trial NCT02432846 reported that local delivery of allogeneic DCs combined with the tyrosine kinase inhibitor sunitinib resulted in a partial response in 30.8% of patients with metastatic renal cell carcinoma. Further, phase II of clinical trial NCT04796194 examines the intratumoral administration of LTX-315, an oncolytic peptide, with systemic anti-PD-1. In phase I, this combination demonstrated an acceptable safety profile and substantial volume reduction in 29% of the patients, and 86% of biopsies had an increase in intralesional CD8^+^ T cells posttreatment (NCT04796194) (188). However, direct comparisons between systemic and localized treatment regimens are still lacking. In the case of peptide vaccines, there are only a few clinical trials investigating the intratumoral administration of peptide/protein vaccines, emphasizing the need for such studies.

While most clinical studies prioritize systemic administration routes for both protein/peptide and DC vaccines, intratumoral delivery has shown a potential to mitigate the immunosuppressive tumor microenvironment. Preclinical evidence supports this approach. A study investigating antigen-pulsed DCs demonstrated that intratumoral administration, when combined with subcutaneous delivery, led to a reduction in regulatory T cell (Treg) populations, decreased TGFβ expression, and increased T cell infiltration within a glioblastoma mouse model. This dual administration strategy exhibited superior results compared to subcutaneous injection alone (189). While the preclinical studies demonstrate promising results, additional clinical investigation is necessary.

### 5.2 Intratumoral immunomodulators

Immunomodulators are a form of immunotherapy that regulate the immune system’s response and encompass cytokine therapy, pattern recognition receptor (PRR) and stimulator of interferon genes (STING) agonists, and vaccine adjuvants (190). STING or PRR agonists like Pam2Cys (toll-like receptor (TLR2), Poly(I:C) or Poly-ICLC (TLR3), monophosphoryl lipid A (TLR4), ADU-S100 (STING), and resiquimod or imiquimod (TLR7/8) when interacting with their respective receptors on tumor or immune cells, can stimulate APCs, macrophages, B and T cells, and the production of various cytokines and chemokines (191, 192). In preclinical studies, intratumorally administered STING agonists have shown promising results across different cancer types (193). In a murine model of metastatic sarcoma, intratumoral administration of STING agonist DMXAA resulted in a 60% reduction in tumor size and prolonged survival. Additionally, the systemic anti-tumor immune response was observed in this metastatic model, resulting in approximately 50% reduction of primary lesions and lung metastases (194). Based on promising preclinical results, several clinical trials involving intratumoral STING agonists were initiated, preferably combined with ICI therapy (**Supplementary Table 2**). For example, when STING agonist MK-1454 was administered intratumorally as monotherapy, complete (CR) or partial responses (PR) in clinical trials for advanced solid tumors were not achieved. However, when combined with ICI, a PR of 24% (6/25) was observed, with reductions in both injected and non-injected lesion sizes (195) (NCT03010176). In a follow-up study involving patients with advanced or metastatic head and neck squamous cell carcinoma, an overall response rate of 50% was noted with this combination treatment (NCT04220866). Additionally, newer STING agonists like BMS-986301, MK-2118, ONM-501, and BMS-986301 hold the potential to enhance our understanding of STING agonists’ role in cancer immunotherapy (**Supplementary Table 1**).

PRR agonists are a significant focus of the clinical trials under discussion here and elsewhere (**Supplementary Table 2**) (196). The lack of tumor specificity and dose-limiting systemic toxicities upon intravenous administration increased interest in intratumoral administration as an alternative approach (197, 198). Various TLR9 activators, including CMP-001, SD-101, tilsotolimod, MGN1703, and CpG, have been investigated primarily in conjunction with ICI therapies to assess their safety, tolerability, and ability to stimulate immune responses within the tumor microenvironment (199). For instance, early data on SD-101 with PD-1 blockade showed increased clinical efficacy with minimal additional toxicity relative to PD-1 blockade alone in advanced melanoma (200). Efficacy of SD-101 is currently being evaluated in combination with PD-1 blockade and radiation therapy in patients with metastatic pancreatic cancer (NCT04050085) and prostate cancer (NCT03007732); and in combination with anti-OX40 antibody in patients with advanced solid tumors (NCT03831295). CMP-001, combined with PD-1 blockade therapy, entered phase II/III study for patients with unresectable or metastatic melanoma (NCT04695977). However, the study was terminated due to business decisions. CMP-001 is being further evaluated in various locally advanced and metastatic cancers (**Supplementary Table 1**). The results of another phase I trial demonstrated that the combination of CMP-001 with PD-1 inhibitor pembrolizumab elicited the best objective response rate (ORR) per RECIST v1.1 criteria of 23.5% (95% CI, 15.5%-33.1%) Moreover, post-progression responders achieved the best ORR of 27.6% (95% CI, 19.0%-37.6%) (NCT02680184) (201). Optimizing the mixture of carefully selected immunomodulators and therapeutics for direct intratumoral combined administration may increase vaccine efficacy. For instance, in a murine model of pancreatic adenocarcinoma, a combination of intratumorally administered TLR agonists (Poly (I:C), R-848, LTA), mannan-BAM, and agonistic anti-CD40 antibodies resulted in a notable 67% decrease in tumor size (202). Likewise, in the bilateral murine colon cancer model, the synergistic effects of this treatment induced systemic immune response, resulting in tumor growth delay and complete tumor regression in a subset of untreated representative metastatic tumors (203).

Besides STING and PRR agonists, bacteria-based therapies are examined in the advanced disease setting. For instance, attenuated *Clostridium novyi*, depleted of its lethal toxin gene, is currently tested for intratumoral administration in clinical trials with systemic ICI treatment (NCT03435952). This vaccine administration has previously demonstrated tumor-specific T-cell induction and decreased tumor size (204) (NCT01924689), supporting the integration of bacterial vaccines amongst potential immunotherapeutic strategies for advanced diseases. Another clinical study with T3P-Y058-739, a genetically modified, live attenuated strain of the bacterium *Yersinia enterocolitica*, alongside ICI treatment, will be evaluated in patients with advanced solid tumors (NCT05120596).

Additionally, various cytokines such as IL-2, IL-12, interferon (IFN)-α, and GM-CSF can trigger anti-tumor immunity or inhibit angiogenesis (75, 76). Their short half-life, low biodistribution, and toxicity limit their practical systemic application (76, 205). However, directly injecting the immunomodulators into the tumor site could optimize their efficacy. In murine melanoma and colorectal cancer models, intratumoral administration of mRNA encoding IFN-α, IL-12, IL-15, and GM-CSF enhanced by systemic administration of anti-PD-1, reduced tumor growth of both primary tumors and metastases (206). Several methods to deliver cytokines directly in the tumors alongside systemic therapy (mostly ICIs) for patients with advanced diseases have been tested, including mRNA vaccines (NCT06249048), adenoviral vector encoding IL-12 (NCT04050085, NCT04006119, NCT02423902), recombinant fusion proteins (NCT06284590), cytokines (NCT01480323, NCT01672450), or plasmids (NCT02493361, NCT04526730).

### 5.3 Intratumoral adoptive cell therapies

Adoptive cell therapies enhance the immune system’s ability to fight cancer by administering genetically engineered or expanded patient immune cells that can specifically target and destroy cancer cells. This approach includes CAR-T therapy, tumor-infiltrating lymphocyte (TIL), NK cell (39, 40), and γδ-T cell therapy, and CAR therapy for cells of innate immunity (e.g., CAR-NK, CAR-NKT, or CAR macrophage). Despite their promising potential, these therapies can face challenges such as antigen escape (207), low infiltration of transferred cells into tumor lesions, and the presence of immunosuppressive mechanisms, including a hostile tumor environment and immunosuppressive cells (208). Furthermore, one of the limitations is their short half-life and cytokine release syndrome upon intravenous administration (209, 210). Local administration may facilitate the infiltration of adoptively transferred cells (40, 208) and mitigate systemic toxicity (211), highlighting additional advantages of intratumoral administration for such cell therapies.

For treating advanced cancers in both clinical and preclinical settings, cell therapies are often combined with approaches that induce oncolysis. These combinations includes an intratumoral administration of zoledronate-pulsed dendritic cells with intravenous T lymphocytes and gemcitabine (212), chemotherapy (213) (NCT02018458), low-dose cisplatin and 5-fluorouracil (214), photodynamic therapy (215), oncolytic viral therapy (216), or intratumoral CD4⁺ Th1 memory cells and cryoablation (NCT00861107). The repeated intratumoral application of CD1c myeloid DC alongside ICI and synthetic saponin-based adjuvant ASO1b, together with systemic low-dose ICI, has demonstrated encouraging results in treating refractory advanced melanoma (217) (NCT03707808). In this phase I trial, 4 patients (50%) obtained complete response (CR) in the injected lesions. Of these, 2 patients obtained an overall CR, and one patient PR. Median progression-free survival (PFS) and overall survival (OS) were 24.1 and 41.9 weeks, respectively (217). Furthermore, autologous DCs injected with an adjuvant booster (Prevnar vaccine) are being studied in patients with unresectable intrahepatic cholangiocarcinoma after standard high-dose external beam radiotherapy (NCT03942328). This approach has shown a favorable safety profile and encouraging signs of efficacy and induction of tumor-specific immunity. Early response data from the five subjects who have completed the protocol showed ORR of 60% (n=3, all partial response) (218). Phase II of the study NCT03942328 will focus on combinations with ICI that could further enhance immunotherapy outcomes. The promising results of integrating cell-based vaccines with other treatments warrant additional clinical investigation to broaden the range of available immunotherapy options.

### 5.4 Intratumoral immune checkpoint inhibitors and monoclonal antibodies

Checkpoint molecules, such as cytotoxic T-lymphocyte associated protein 4 (CTLA-4) and programmed cell death protein 1/programmed cell death ligand 1 (PD-1/L1), maintain self-tolerance and prevent any autoimmune reactions by modulating the activity of T cells (78). Nevertheless, tumor cells can exploit checkpoint molecules to evade immune responses. Thus, blocking these checkpoints can immunity against cancer. For example, an antagonistic monoclonal antibody anti-CTLA-4 releases the inhibition of APC activity mediated by the interaction between CTLA-4 on Tregs and CD80/86 on APC. Additionally, anti-PD-1 or anti-PD-L1 antibodies reverse the negative interaction between tumor cells and T cells, thereby stimulating their activity (77, 78). Successful checkpoint inhibitor therapy depends on the presence of pre-existing anti-tumor immunity (65). Some studies report poor treatment responses to checkpoint blockade due to impaired antigen presentation, loss of neoantigens, insufficient T cell infiltration, or inhibition of T cell killing activity in an immunosuppressive tumor microenvironment (TME) (219). Furthermore, treatment with systemic monoclonal antibodies, including checkpoint inhibitors, is accompanied by immune-related adverse events (220, 221)(e.g., NCT01844505, NCT02142738, NCT02477826) due to their long serum half-life enabling the interaction with various cells. High molecular weight also mitigates the intratumoral bioavailability (197, 221). Some of these obstacles, particularly immunosuppressive TME, can be targeted with intratumoral administration (222). Additionally, combinations of checkpoint inhibitors with other immunomodulatory approaches to overcome immune evasion mechanisms, or with cytostatic drugs targeting cancer cell growth, immortality, angiogenesis, or genome instability, have been introduced (223).

Current clinical trials focus on a combination of *in situ* vaccination and systemic treatment, mainly targeting immune checkpoints. The reason is a synergy observed between local immunostimulatory therapies and systemic checkpoint inhibitors (224) and approval of numerous checkpoint inhibibtors for systemic administration, such as antagonistic CTLA-4 (e.g., ipilimumab and tremelimumab) and anti-PD-1/L1 antibodies (e.g., pembrolizumab, atezolizumab, nivolumab, cemiplimab, durvalumab, avelumab), alone or in combination with other therapeutic interventions. Among novel checkpoint molecules, such as lymphocyte activation gene 3 protein (LAG-3) and T cell immunoglobulin mucin-3 (78, 225), systemic dual inhibitor of PD-1 and LAG-3, Opdualag, is currently the only one authorized by the FDA for clinical use for unresectable or metastatic melanoma. To date, intratumoral checkpoint blockade has been combined with approaches that stimulate the formation of cytotoxic T cells, such as anti-CD40 monoclonal antibody, and hypofractionated radiotherapy (226); oncolytic viral therapy (227, 228) (NCT04725331); and chemotherapy (229). Furthermore, to counteract the resistance that may develop against immune checkpoint inhibitors (230–232), combination therapies with immunostimulatory agents such as BO-112 (233), CMP-001 (CMP-001-001; NCT02680184), SD-101 (234), and bacteria *Fusobacterium nucleatum* (235), or targeted therapy such as VEGF (236) and CDK4/6 inhibitor (237), have been introduced, underscoring the necessity for continued immunostimulation. Additionally, CD40 is a promising target for immune checkpoint therapies. CD40 agonistic monoclonal antibodies stimulate cells of both the innate and adaptive immune systems, including macrophages, neutrophils, and DCs (238). Currently, several clinical trials in various stages are investigating anti-CD40 therapies in combination with irreversible electroporation (NCT06205849) and pembrolizumab (NCT02706353, NCT02988960). One completed trial examined the combination of anti-CD40 with a TLR agonist (NCT03831295).

Currently, no checkpoint inhibitor has been approved by either the FDA or EMA for intratumoral administration in the advanced disease setting. However, clinical studies on intratumoral administration of checkpoint inhibitors in advanced tumors, especially with combination therapies, are ongoing (e.g., NCT03707808). For instance, intratumoral checkpoint blockade has been combined with oncolytic viral therapy (227) and chemotherapy (229). While studies support the preference for combined therapies to enhance immune checkpoint blockade efficacy, the outcomes of intratumoral administration combined with cytotoxic therapies for patients with advanced and metastatic disease are yet to be fully explored. Current evidence regarding potential combinations for both intratumoral and systemic administration could expedite further research.

## 6 Timing of intratumoral immunotherapies combined with other treatment modalities

Understanding and optimizing the timing of intratumoral immunotherapy administration alongside other therapeutic approaches is essential for maximizing therapeutic efficacy and improving patient outcomes. Chemotherapy and radiotherapy can significantly affect the viability and function of immune cells, thus, the ideal timing for each combination should be studied thoroughly (239, 240). It should also be noted that many patients have undergone previous treatments, which may impact the function of therapeutic combination. While the timing for some systemic combination therapies for the treatment of advanced cancers has been explored (**Supplementary Table 2**), the timing for intratumorally administered therapies may differ significantly from systemic administration. For instance, there may be leakage of therapeutics into nearby tissues or systemic circulation following local injection (241). However, localized delivery of chemotherapeutics can potentially reduce systemic adverse effects often connected with higher therapeutic dose. Leakage can be mitigated through specific injection techniques or by injection of various sites of a tumor, as well as by a needle type (81, 242, 243). This allows for more flexibility in timing for combined therapies.

ICIs are often combined with prior chemotherapeutic intervention, while targeted therapies typically precede chemotherapy or radiotherapy (**Supplementary Table 1**). Although administering checkpoint inhibitors before chemotherapy regimens may be unconventional and not fully aligned with the potential induction of ICD by chemotherapy (104–107, 109, 244), there are promising results. For instance, results reported by Szabados et al. demonstrated the benefits of this schedule. Patients with metastatic urothelial carcinoma who received initial treatment with ICIs followed by chemotherapy had a better response rate (64%) compared to those who received chemotherapy before ICI treatment (21%) (245). However, studies on lung cancer and advanced esophageal squamous cell carcinoma point to improved treatment efficacy when immunotherapy is applied several days after chemotherapy (246). Additionally, recent studies have explored the role of circadian signals in cancer development, immune system recognition, and the effectiveness of immunotherapies (247, 248). For example, early systemic administration of checkpoint inhibitors has been shown to extend patient survival four times longer than a late-day administration (249, 250).

In the case of cell-based therapies, chemotherapy-induced lymphodepletion prior to cell therapies like CAR-T has been shown to enhance CAR-T proliferation. This approach not only facilitates an early treatment option for solid tumors but also addresses the prolonged development and infusion timelines associated with CAR-T therapy (251). In the context of targeted therapy and chemotherapy combinations, approved treatments typically involve administering monoclonal antibodies, such as those targeting EGF/EGFR, tyrosine-protein kinase erbB-2 receptor, or VEGF/VEGFR, prior to cytotoxic therapy. However, in patients with non-small cell lung cancer, pretreatment with an anti-VEGF antibody (bevacizumab) has been found to hinder the delivery of chemotherapeutic drugs (252).

These findings underscore the variability in treatment outcomes across different cancer types and highlight the critical need for additional research in this area. Determining the optimal timing for administering these therapies is crucial for integrating them into standard care. This decision must account for factors such as the time required for the immune system to develop a specific response to the antigen, the necessity of multiple doses, and potential interactions with concurrent therapies. Furthermore, time schedules are often not reported in clinical trials or not fully described when treatments are administered on the same day (e.g., NCT01672450, NCT02493361, NCT04220866) (**Supplementary Table 2**). It cannot be implied whether concurrent or non-simultaneous administration of combination therapies is necessary. Additionally, there are currently no clinical trials examining different time schedules of the same treatment for advanced cancers emphasizing the urgent need for such studies.

## 7 Clinical trials

The landscape of cancer treatment has undergone a significant transformation with the introduction of intratumoral immunotherapy, particularly for locally advanced and metastatic cancers. Numerous clinical trials examining the safety and efficacy of combinations of intratumoral immunotherapy with other treatment modalities have been conducted to date. These mainly include intratumoral oncolytic viral therapy or PRR agonists in combination with systemic checkpoint inhibitors (**Supplementary Table 1**).

Designing clinical trials can be highly complex, often serving as a final treatment option when other therapies have failed, particularly in advanced disease cases. The FDA’s approval of metastasis-free survival (MFS) as an endpoint for non-metastatic prostate cancer marks a significant advancement (253). This decision reflects shortcomings in using PFS as an endpoint for metastatic disease, as PFS fails to provide insights into metastatic activity, which is the primary cause of death (254). Recently, numerous clinical trials have adopted MFS as either a primary or secondary outcome for prostate (NCT05352178, NCT04641078, NCT03569241, NCT01341652), nasopharyngeal (NCT03290820), colorectal (NCT00643877), and breast cancer (NCT04278469, NCT02448576), and melanoma (NCT06157099). However, there are currently no trials utilizing MFS outcomes for the advanced disease setting.

Most studies on the combination of intratumoral immunotherapy with other therapies are currently in phases I/II (**Supplementary Table 1**). NCT04695977 entered phase II/III but has been recently terminated due to business decisions. In this study, vaccinia virus Pexa-Vec was administered as 3 bi-weekly intratumoral injections, followed by protein kinase inhibitor sorafenib at week 6. The median TTP was 2.0 months (95% CI: 1.77, 2.96) and 4.2 months (95% CI: 2.92, 4.63); ORR was 19.2% (45 patients) and 20.9% (47 patients); and DCR was 50.0% (117 patients) and 57.3% (129 patients) in the Pexa-vec plus sorafenib and sorafenib arms, respectively. The median OS was 12.7 months (95% CI: 9.89, 14.95) in the Pexa-vec plus sorafenib arm and 14.0 months (95% CI: 11.01, 18.00) in the sorafenib arm, which led to early termination of the study (255). This underscores how essential it is to thoroughly evaluate the safety, specificity, and efficacy to successfully navigate this innovative path in immunotherapy. The primary goal of these comprehensive strategies is to choose a treatment regimen that enhances effective, long-lasting, and tumor-specific immunity in cancer patients, thereby extending their survival. Moreover, ongoing advancements allow for investigating potential new therapeutic approaches, as outlined in the final chapter.

## 8 Future directions in the treatment of advanced cancer

### 8.1 Photoactivated therapy

Photoactivated therapy, also known as photodynamic therapy (PDT), represents a promising approach in the targeted treatment of advanced cancers. This therapy utilizes specific photosensitizing agents that preferentially accumulate in tumor tissues and, upon exposure to light of a particular wavelength, generate reactive oxygen species (ROS). These ROS lead to localized tumor cell destruction, minimizing damage to surrounding healthy tissue and resulting in fewer side effects (256, 257). Furthermore, PDT can induce immunogenic cell death, thereby activating the immune system to recognize and target residual cancer cells and lesions, which may enhance antitumor immunity (258, 259).

The targeted approach of PDT makes it particularly suitable for localized treatment of multidrug-resistant and clinically challenging tumors (260), as well as advanced and metastatic tumors (261), especially when combined with other modalities (262). In future scenarios, PDT could be effectively integrated with intratumoral immunotherapy to strengthen local immune responses and improve patient outcomes in cases of locally advanced and metastatic cancer.

As technological advancements enable deeper tissue penetration and more precise light delivery, PDT could play a crucial role in enhancing targeted drug delivery. These developments align with current efforts in optimizing intratumoral immunotherapy for challenging malignancies, highlighting PDT’s potential as a complementary therapeutic modality in the management of advanced cancer.

### 8.2 Neoadjuvant setting

Therapeutic options for cancer diseases have undergone significant transformation in recent years, particularly neoadjuvant immune checkpoint inhibitors. Neoadjuvant cancer therapy, administered usually before surgery, traditionally aims to shrink tumors to facilitate surgical resection (263). However, the innovative application of ICIs in the neoadjuvant setting has introduced a paradigm shift, offering improved resectability and enhanced systemic anti-tumor immunity. This dual approach leverages the primary tumor as a source of antigens, thereby inducing an immune response capable of targeting and eliminating dormant tumor cells or distal micrometastases, which are often connected with post-surgical relapse (264).

**Figure 3:**
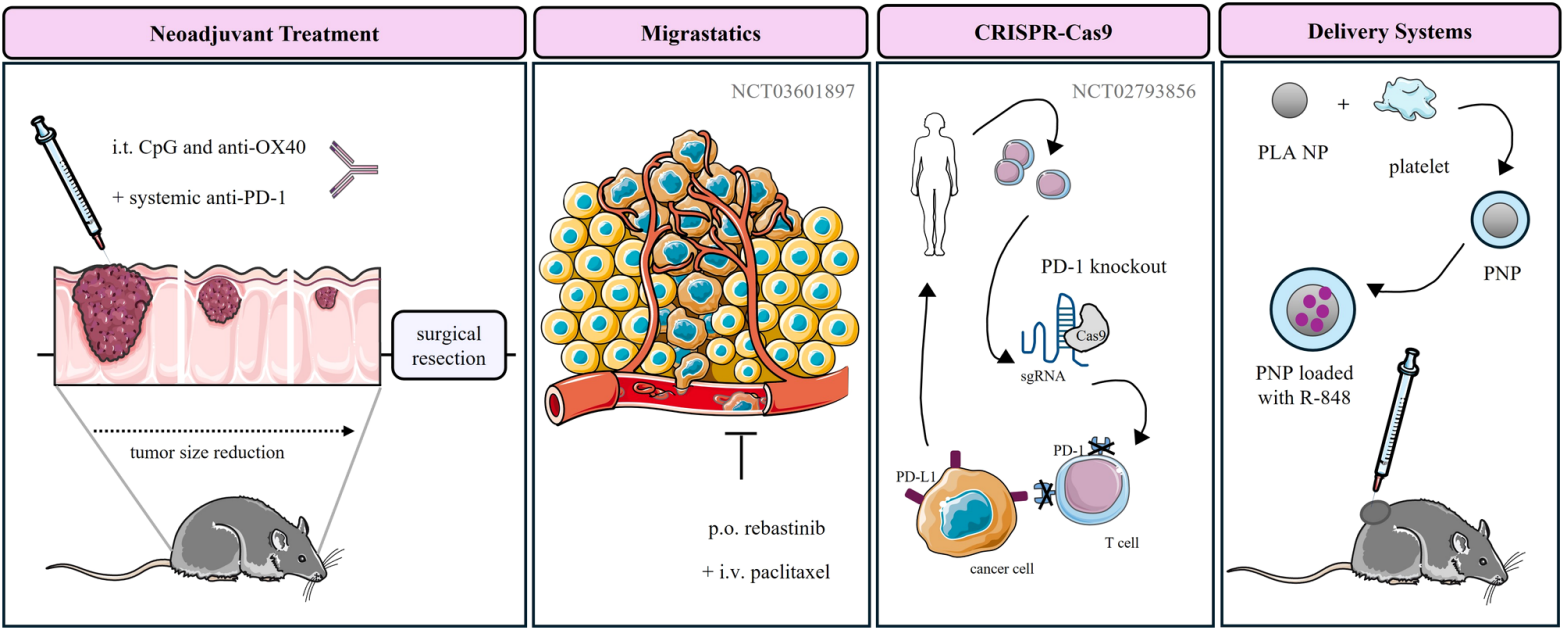
Novel therapeutic approaches for the treatment of advanced cancers. Up to date, intratumoral neoadjuvant immunotherapy combined with other treatment approaches has only been studied preclinically. Agents with anti-migratory activity, such as rebastinib, are currently being investigated in clinical trials through systemic administration. Additionally, the CRISPR-Cas9 gene editing tool is being utilized to knock out the PD-1 gene in patients’ T cells. However, no clinical trials have yet examined a combination approach. Novel delivery systems, such as platelet membrane-coated nanoparticles, have only been studied in animal models. CpG, cytosine-phosphate-guanine; PD-1, programmed cell death protein 1; PD-L1, programmed death ligand 1; sgRNA, single guide RNA; Cas9, Clustered regularly interspaced short palindromic repeats-associated protein 9; NP, nanoparticle; PLA, polylactic acid; PNP, platelet coated nanoparticle; R-848, resiquimod 848.

Recent clinical trials have shown promising advancements in using neoadjuvant ICIs across various cancers. In advanced melanoma, the SWOG S1801 trial demonstrated that event-free survival at 2 years was 72% in the neoadjuvant-adjuvant pembrolizumab group compared to 49% adjuvant therapy, establishing neoadjuvant immunotherapy as a new standard of care (265). For non-small cell lung cancer, results from clinical trials have shown favorable pathologic response rates with minimal adverse events when using ICIs like nivolumab in a neoadjuvant setting (266). Neoadjuvant ICIs in breast cancer have also demonstrated significant improvements in pathologic complete response rate and event-free survival in clinical trials (267). Similarly, neoadjuvant immunotherapy with nivolumab and ipilimumab in locally advanced mismatch repair-deficient colon cancer has shown groundbreaking results (268). Additionally, the intratumoral neoadjuvant treatment consisting of CpG, a TLR9 agonist, and anti-OX40 achieved low toxicity and enhanced systemic response in a murine model of metastatic colorectal and breast cancer. A short break between the intratumoral immunotherapy and surgical resection was essential for the treatment efficacy (**Figure 3**) (269). These examples of clinical trials highlight the potential of neoadjuvant immunotherapy to improve surgical outcomes and survival rates across different cancer types. Several neoadjuvant intratumoral immunotherapy trials have been underway as well, including virotherapy approaches, TLR agonists, gene therapies, or cell-based vaccines (80).

The future of neoadjuvant immunotherapy has promising research and ongoing clinical trials, which may uncover innovative treatment combinations and strategies. With such knowledge of tumor immunology and immune checkpoint pathways, we anticipate even more effective and personalized neoadjuvant therapies, leading to better surgical outcomes, reduced relapse rates, and improved overall survival for cancer patients.

### 8.3 Migrastatics

Although the research focuses mainly on targeting the proliferative capacity of cancer cells and generating antiproliferative and cytostatic drugs, the inhibition of cancer cell motility is gaining increased interest as the presence of metastasis represents a significant challenge for today’s oncology (147). Migrastatics, a term first proposed by Gandalovicova et al., 2017, represent a group of drugs that aim to inhibit the dissemination of cancerous cells to distant sites. Cytostatic drugs exert high cytotoxic stress on cancer cells, often leading to the selection of resistant cell populations over time. Since migrastatics do not kill cancer cells directly but rather inhibit their ability to migrate and spread, there is less selective pressure for the development of drug-resistant mutations. Although drugs with anti-migratory activity do not aim to reduce primary lesions, the inhibition of cancer cell motility lowers the number of resistant cells within the tumor (133).

To date several candidates targeting actin polymerization, actomyosin contractility, tropomyosin, myosin, and cAMP-dependent, cGMP-dependent, and protein kinase C (AGC) kinases or a stabilization/destabilization of actin cytoskeleton have been identified (133), including novel and repurposed drug targets. These include rebastinib (**Figure 3**) (270), paclitaxel, docetaxel, metformin, tamoxifen, mitotam, and voloxicimab (271). Furthermore, the inhibition of epithelial-mesenchymal (EMT) or mesenchymal-epithelial transition (MET) (271–273), matrix metalloproteinases (MMP), cell adhesion (134), or the SDHB subunit of oxidative phosphorylation complex II has been described to reduce cancer cell invasiveness and metastasis (274).

In the case of advanced cancers, migrastatics in combination with antiproliferative drugs are believed to achieve great efficacy (133). Currently, there are two completed clinical trials (NCT03717415; NCT03601897) and one clinical trial (NCT02824575) terminated by a pharmaceutical company, examining the efficacy of rebastinib in combination with chemotherapy for the treatment of metastatic disease. Although intratumoral administration of migrastatics may reduce the side effects upon continuous treatments (65), including the mitigation of motility or cytokinesis of healthy cells (133), lower therapeutic doses of combination therapy (65), and increase sensitivity to mitosis-targeted drugs (133), it can be speculated that circulating tumor cells may be targeted with a systemic approach. A combination of drugs with anti-migratory activity and reduced selective pressure for drug-resistant mutations, along with other treatments, requires further clinical investigation in both systemic and intratumoral settings.

### 8.4 CRISPR-Cas9

CRISPR-Cas9, clustered regularly interspaced short palindromic repeats/associated protein 9, is a gene editing tool with a potential application in CAR-T and TIL adoptive cell therapies, the generation of cancer animal models, and drug screening (275, 276). The development of CAR-T therapy from each patient is time-consuming, excluding those patients with metastatic disease. By incorporating CRISPR-Cas9 to generate CAR-T cell therapy from healthy donors, i.e., shifting the therapy from autologous to allogeneic CAR-T cells, a shortened and better quality manufacturing period, lower treatment cost, and a higher number of T cells could be achieved (277). Besides the genetic modification of CAR specificity, additional alterations, such as a deletion of PD-1 (278) or LAG-3 (279), or the repair of KRAS oncogenic mutations (280) can be introduced.

To date, several clinical trials have examined the safety and efficacy of CRISPR-Cas9-engineered CAR-T cells for the treatment of advanced cancer. For instance, a phase I clinical trial examined the safety of CAR-T PD-1 knockout therapy in patients with metastatic non-small cell lung cancer after chemotherapy treatment (NCT02793856) (**Figure 3**). The majority of clinical trials examine the systemic administration, including NCT04417764, NCT04976218, and NCT05812326, although the development of autoimmune reactions against donor TCR and HLA molecules, such as host versus graft response and graft versus host disease can be expected (281). Intratumoral administration of CRISPR-Cas9-modified cell-based therapies is scarce, including the injection of mRNA-transfected c-Met-CAR T cells in the treatment of metastatic breast cancer (NCT01837602).

Although CRISPR-Cas9 is a promising gene-editing tool for oncologic application, local administration in both early stage and advanced disease settings requires further examination.

### 8.5 Delivery systems

Besides the local administration of therapeutic agents into tumor lesions, approaches to enhance drug localization and distribution specifically to tumor sites have also been investigated. Ongoing advancements in biomaterial development focus on enhancing physicochemical properties and particle size to improve drug retention, ensure uniform distribution within lesions, regulate drug release, and enhance drug solubility (282–284). To date, several drug nano-delivery systems have been developed and include organic nanoparticles (e.g., polymersomes, polymeric nanoparticles and micelles, liposomes, and lipid nanoparticles) (283, 285) and inorganic nanoparticles (e.g., gold and iron particles, hydrogels, and silica), peptide and antibody-drug conjugates (285, 286), extracellular vesicles (e.g., apoptotic bodies, exosomes, and microvesicles) (287), targeted protein degradation systems (e.g., LYTAC and PROTACS) (288), cell or cell-membrane coated nanoparticles (e.g., erythrocytes, platelets, macrophages, neutrophils, leukocytes or tumor cells) (289, 290) and oncolytic virus-based delivery systems (285, 291). Additionally, novel transdermal patches (292), hydrogels (292), or sprayable gels (293) are under investigation.

Particularly noteworthy are peptide and antibody-drug conjugates, which ensure reduced toxicity and precise tumor targeting (285), and cell-based delivery systems utilizing organic and inorganic nanoparticles coated with cell membranes to evade immune recognition (289, 290). To date, several delivery systems have been approved for the systemic treatment of advanced cancers, including, Myocet (liposome-encapsulated doxorubicin), Abraxane (albumin-bound paclitaxel), Onivyde (liposomal topoisomerase inhibitor), Kadcyla (trastuzumab-DM1 conjugate), Enhertu (trastuzumab-deruxtecan conjugate), Padcev (enfortumab-vedotin conjugate) or Trodelvy (sacituzumab-govitecan conjugate). Systemic toxicities associated with these treatments can be mitigated by local administration, which lowers systemic exposure, increases drug concentration, and prevents drug leakage into the bloodstream. Factors such as particle size, charge, and injection rate significantly influence nanoparticle distribution within the tissue (294).

Although no intratumoral delivery system for intratumoral application has been approved yet, several studies examined the encapsulation of immunotherapies for local administration. For instance, local administration of CpG and anti-PD1 antibody DNA nano-cocoon after resection of primary tumor into tumor bed inhibited the disease recurrence and metastasis generation (295). Similarly, silica-zinc oxide micro-rosettes loaded with doxorubicin and Poly(I:C) were reported to reduce primary tumor and metastases growth (296). Furthermore, inhibition of the growth of primary tumors and metastases has been achieved upon intratumoral treatment with platelet membrane-coated nanoparticles loaded with R848 (297) (**Figure 3**), and polymeric nanoparticles loaded with antigen peptides (298). In conclusion, continued research and development in targeted drug delivery systems are essential to improve the efficacy and safety of cancer treatments.

## 9 Conclusion

Despite significant advancements, locally advanced and metastatic cancers remain a critical medical challenge. Conventional anticancer treatments and therapeutic approaches, though effective in certain instances, frequently do not adequately enhance overall patient outcomes and survival rates for those with advanced disease. Intratumoral immunotherapy offers a promising alternative with fewer side effects, lower costs, and reduced toxicity. T-VEC monotherapy is the only intratumoral immunotherapy approved for the treatment of unresectable melanoma lesions. The future of cancer treatment lies in the development of combination therapies that induce immunogenic cell death and target multiple hallmarks of cancer. These approaches can potentially enhance the therapeutic response and reduce the likelihood of resistance. Emerging strategies such as migrastatics, CRISPR-Cas9- modified cell therapies, and advanced drug delivery systems represent promising avenues for the treatment of advanced cancers.

Future research should focus on optimizing these innovative treatments and integrating them into standard care. This includes further investigation into the timing and sequencing of combination therapies, exploring new targets and mechanisms of action, and improving drug delivery systems to enhance specificity and reduce toxicity. Additionally, personalized medicine approaches, leveraging genomic and molecular profiling, will be crucial in tailoring treatments to individual patients, maximizing efficacy, and minimizing adverse effects. By addressing the limitations of current therapies and exploring new frontiers, we can move closer to achieving effective, long-term control of locally advanced and metastatic cancers, providing new hope for fighting cancer.
