# Supplementary material for: Injecting hope: the potential of intratumoral immunotherapy for locally advanced and metastatic cancer
Source: Front Immunol. 2025 Jan 9;15:1479483. doi: 10.3389/fimmu.2024.1479483 (PMC11754201; doi:10.3389/fimmu.2024.1479483)
Supplement: Supplementary file 1 [file DataSheet1.pdf]

1 Supplementary Tables

Supplementary Table 1| **Clinical trials examining the safety and efficacy of intratumoral immunotherapies combined with other treatment approaches.**

		Trial Number	Study Status	Primary Outcome	Study Results	Condition	Phase	Study Design	Start Date
Checkpoint Inhibitors and Monoclonal Antibodies	Checkpoint Inhibitors	**NCT06413095**	Completed	immune cell biomarkers	NO	head and neck squamous cell carcinoma, soft tissue sarcoma	early phase I	i.t. pembrolizumab (anti-PD-1), i.t. MK-0482 (anti-ILT3), and pembrolizumab (anti-PD-1)	2022
		**NCT04370587**	Recruiting	DLT, TEAE,	NO	advanced and metastatic solid tumors	phase I/II	i.t. T3011 (herpes simplex virus encoding anti- PD-1 and IL-12) and i.v. pembrolizumab (anti-PD-1)	2020
		**2020-000505-80** **Belgium**	Ongoing	AE, DLT,	NO	metastatic/advanced solid cancer	phase I/IIa	i.t. BT-001 (oncolytic vaccinia virus expressing anti-CTLA-4 and GM-CSF) and i.v. pembrolizumab (anti-PD-1)	2020
		**NCT04735978**	Active, not recruiting	DLT, TEAE, SAE, RP2D	NO	advanced solid cancer	phase I	i.t. RP3 (herpes simplex virus expressing anti-CTLA-4, CD40L, and 4-1BBL) and i.v. nivolumab (anti-PD-1)	2020
		**NCT03707808**	Completed	TEAE	NO	solid cancer, soft tissue metastases	phase I	i.t. ipilimumab (anti-CTLA-4) and avelumab (anti-PD-L1), i.t. autologous DCs, and i.v. nivolumab (anti-PD-1)	2018
		**NCT01672450**	Completed	AE	NO	unresectable melanoma	phase I	i.t. ipilimumab (anti-CTLA-4) and i.t. IL-2	2012

	Monoclonal Antibodies	**NCT06455605**	Not yet recruiting	Safety and efficacy	NO	recurrent glioblastoma	phase I/II	i.t. D2C7 (antibody-drug conjugate) and i.v. 2141 V11 (anti-CD40)	2024
		**NCT06205849**	Recruiting	safety and tolerability	NO	advanced pancreatic cancer	phase I	i.t. mitazalimab (anti-CD40) irreversible electroporation	2024
		**NCT04547777**	Recruiting	DLT	NO	stage III and IV glioma	phase I	i.t. 2141-V11 (anti-CD40) and 2141-V11 (antibody-drug conjugate)	2021
		**NCT03831295**	Completed	DLT	NO	advanced and metastatic solid cancer	phase I	i.t. and i.v. BMS 986178 (anti-OX40) and i.t. SD-101 (TLR9 agonist)	2019
		**NCT02988960**	Active. not recruiting	MTD, RP2D, AE	NO	advanced solid cancer	phase I	i.t. ABBV-927 (anti-CD40) and i.v. ABBV-181 (anti-PD-1)	2017
		**NCT02706353**	Active, not recruiting	MTD, RP2D, ORR	NO	metastatic melanoma	phase I/II	i.t. APX005M (anti-CD40) and i.v. pembrolizumab (anti-PD-1)	2017
Adoptive cell therapies		**NCT06478459**	Not yet recruiting	MTD, DLT	NO	advanced pancreatic cancer	early phase I	i.t. NKG2D CAR-NK and i.v. NKG2D CAR-NK	2024
		**NCT04385173**	Recruiting	AE, MTD, OS, PFS	NO	recurrent glioblastoma	phase I	i.t. or intracerebroventricularly B7-H3 CAR-T and p.o. temozolomide	2022

		**NCT05605197**	Recruiting	AE	NO	advanced solid cancer	phase I	i.v. cyclophosphamide followed by i.v. U87 CAR-T, intraartery U87 CAR-T, and i.v. IL-2. Possibility to select i.t. injection.	2022
		**NCT04214392**	Recruiting	DLT	NO	recurrent glioblastoma	phase I	intracranial intratumoral or intracavitary combined with intracranial intraventricular CAR-T targeting chlorotoxin domain	2020
		**NCT03740256**	Recruiting	DLT	NO	advanced HER2-positive cancer	phase I	i.t. CAdVEC (oncolytic adenovirus) and HER2 CAR-T	2020
		**NCT00861107**	Completed	toxicity	NO	metastatic cancer	phase I/II	i.d. AlloStim-7, followed by cryoablation, i.t. AlloStim-7, and i.v. AlloStim-8 or AlloStim-9 (T-cell therapy)	2009
		**NCT00861107**	Completed	toxicity	NO	metastatic cancer	phase I/II	i.d. AlloStim-7, followed by cryoablation, i.t. AlloStim-7, and i.v. AlloStim8 or AlloStim-9 (T cell therapy)	2009
Cancer Vaccines	Dendritic Cell Vaccines	**NCT03942328**	Recruiting	DLT, PFS	NO	unresectable liver cancer	phase I/II	i.t. DCs, i.m. Pneumococcal 13-valent conjugate vaccine with/without i.v. atezolizumab (anti-PD-L1), i.v. bevacizumab (anti-VEGF)	2019

	**NCT03546361**	Active, not recruiting	MTD, ORR, MAD	NO	stage IV non-small cell lung cancer	phase I	i.t. Ad-CCL21-DC (DCs transfected with adenoviral vector encoding CCL2) and i.v. pembrolizumab (anti-PD-1)	2019
	**NCT03707808**	Completed	TEAE	NO	solid cancer, soft tissue metastases	phase I	i.t. autologous DCs, i.t. ipilimumab (anti-CTLA-4) and i.t. avelumab (anti-PD-L1), and i.v. nivolumab (anti-PD-1)	2018
	**NCT02432846**	Completed	OS	YES (OS for high risk 323 days, PFS 254 days, CR 7.7%, PR 30.8%)	metastatic renal cell carcinoma	phase II	i.t. intuvax (DCs) with i.v. sunitinib (kinase inhibitor)	2015
	**2014-004510-28**<br><br>**Sweden, Czechia, Hungary, Latvia, Great Britain, Estonia**	Completed	OS	YES<br><br>(OS, Intuvax and sunitinib, intermediate-risk, 1270 days)	metastatic renal cell cancer	phase II	i.t. intuvax (DCs) with i.v. sunitinib (kinase inhibitor)	2015
	**NCT02018458**	Completed	safety	YES (SAE 30%)	locally advanced breast cancer	phase I/II	i.t. DCs and chemotherapy, followed by resection, radiotherapy, and s.c. DCs	2014
Viral Oncolytic Vaccines	**NCT06311578**	Recruiting	DLT, AE	NO	advanced solid tumors	phase I	i.t. JNJ-87704916 (herpes simplex virus) and cetrelimab (anti-PD-1) (administration not specified)	2024

**NCT06253598**	Not yet recruiting	ORR	NO	advanced hepatocellular carcinoma	phase II	i.t. H101 (adenovirus), p.o. lenvatinib (kinase inhibitor), and i.v. tislelizumab (anti-PD-1)	2024
**NCT05155332**	Recruiting	DLT, MTD	NO	advanced and metastatic solid tumors	phase I	i.t. BI 1831169 (vesicular stomatitis virus) and/or i.v. ezabenlimab (anti-PD-1)	2022
**NCT05303090**	Recruiting	DLT, MTD, AE	NO	advanced pancreatic cancer	phase I	i.t. H101 (adenovirus), i.v. tislelizumab (anti-PD-1), and p.o. lenvatinib (kinase inhibitor)	2022
**NCT04197882**	Active, not recruiting	ORR, PR, CR, pCR	NO	advanced melanoma	phase I	i.t. OrienX010 (herpes simplex virus) and i.v. toripalimab (anti-PD-1), followed by surgical resection and i.v. toripalimab (anti-PD-1)	2019
**NCT03172819**	Completed	DLT	NO	advanced solid cancer	phase I	i.t. OBP-301 (adenovirus) and i.v. pembrolizumab (anti-PD-1)	2017
**NCT03252808**	Active, not recruiting	DLT	NO	unresectable pancreatic cancer	phase I	i.t. HF10 (herpes simplex virus) and p.o. TS-1 (chemotherapy)	2017
**NCT03153085**	Completed	BORR	NO	unresectable and metastatic melanoma	phase II	i.t. HF10 (herpes simplex virus) and i.v. ipilimumab (anti-CTLA-4)	2017

**2016-000085-32**<br><br>**Italy**	Completed	DLT, SAE, ORR	YES<br><br>(Phase IIa, SAE 80%)	advanced hepatocellular cancer	phase I/IIa	i.t. Pexastimogene devacirepvec (poxvirus) and i.v. nivolumab (anti-PD-1)	2017
**NCT02779855**	Active, not recruiting	MTD, RP2D, pCR	NO	triple-negative breast cancer	phase I/II	i.t. Talimogene laherparepvec with neoadjuvant i.v. paclitaxel	2017
**NCT02798406**	Completed	ORR	YES (10.4% ORR) (1)	recurrent glioblastoma and gliosarcoma	phase II	i.t. DNX-2401 (adenovirus) and i.v. Pembrolizumab (anti-PD-1)	2016
**NCT02509507**	Completed	DLT, ORR, TEAE (SAE for colorectal cancer 30%)	YES (ORR for TNBC 16.7%, SAE 38.9%)	liver metastases	phase I/II	i.t. talimogene laherparepvec (herpes simplex virus) and i.v. pembrolizumab (anti-PD-1)	2016
**NCT02307149**	Completed	CR, PR	NO	advanced melanoma	phase I	i.t. CAVATAK (coxsackievirus) and i.v. ipilimumab (anti-CTLA-4)	2015
**NCT02562755**	Completed	ORR	YES (ORR 19.2%, SEA 53.67%)	advanced hepatocellular carcinoma	phase III	i.t. Pexa-Vec (vaccinia virus expressing GM-CSF) in combination with p.o. Sorafenib (kinase inhibitor)	2015
**NCT02045589**	Completed	AE and RP2D	NO	advanced pancreatic cancer	phase I	i.t. VCN-01 (adenovirus expressing hyaluronidase) in combination with i.v. gemcitabine and abraxane	2014

	**NCT02272855**	Completed	BORR	NO	unresectable and metastatic melanoma	phase II	i.t. HF10 (herpes simplex virus) and i.v. ipilimumab (anti-CTLA-4)	2014
	**NCT01740297**	Completed	DLT, ORR	YES (CR 13.3%, PR 25.5%)	unresectable melanoma	phase I/II	i.t. Talimogene laherparepvec (herpes simplex virus) and i.v. ipilimumab (anti-CLTA-4)	2013
	**2006-002190-49** **Great Britain**	Completed	Efficacy	NO	advanced head, neck, and esophageal cancer	phase II	i.t. Reolysin (reovirus) and low-dose radiotherapy	2006
Viral Vaccines	**NCT05859074**	Recruiting	AE, SAE	NO	advanced cancer	phase I	i.t. MQ719 (vaccinia virus expressing Flt3 and OX40L) and i.v. pembrolizumab (anti-PD-1)	2023
	**NCT00669734**	Active, not recruiting	MTD, DLT, AE	NO	locally advanced pancreatic cancer	phase I	i.t. or s.c. falimarev (fowlpox encoding CEA, MUC-1, and TRICOM), s.c. inalimarev (vaccinia virus encoding CEA, MUC-1, TRICOM), and sargramostim (GM-CSF)	2010
Viral Repurposed Vaccines	**NCT03990493**	Not yet recruiting	TEAE	NO	advanced melanoma	phase I	i.t. PV-001-DV (dengue virus) and i.v. autologous DCs	2024
	**NCT06229392**	Recruiting	DLT	NO	breast cancer	phase I	i.t. influenza vaccine and chemotherapy	2024

	Peptide Vaccines	**NCT04796194**	Active, not recruiting	ORR, OS, CBR	NO	advanced melanoma	Phase II	i.t. LTX-315 (oncolytic peptide) and i.v. pembrolizumab (anti-PD-1)	2021
Immunomodulators	Bacterial Therapies	**NCT03435952**	Active, not recruiting	MTD	NO	refractory solid cancer	phase I	i.v. pembroliyumab (anti-PD-1) followed by i.t. Clostridium Novyi-NT and i.v. doxycycline	2024
		**NCT05120596**	Recruiting	RP2D, safety	NO	advanced solid cancer	phase I/II	i.t. T3P-Y058-739 (bacterium *Yersinia enterocolitica*) and i.v. pembrolizumab (anti-PD-1)	2022
	TLR/STING Agonists	**NCT06410703**	Not yet recruiting	DLT, MTD	NO	unresectable and metastatic solid cancer	phase I/II	i.t. CAN1012 (TLR7 agonist) and i.v. toripalimab (anti-PD-1)	2024
		**NCT05980598**	Recruiting	mPR	NO	advanced head and neck squamous cell carcinoma	phase II	i.t. TransCon TLR7/8 agonist and i.v. TransCon IL-2 β/γ	2023
		**NCT06022029**	Recruiting	TEAE, DLT, SAE	NO	advanced solid cancer	phase I	i.t. ONM-501 (STING agonist) and i.v. cemiplimab (anti-PD-1)	2023
		**NCT05081609**	Recruiting	RP2D, MTD, SAE	NO	locally advanced and metastatic solid cancer	phase I/II	i.t. TransCon TLR7/8 agonist and i.v. TransCon IL-2 β/γ	2022

**NCT05265650**	Recruiting	AE/SAE	NO	metastatic non-small cell lung cancer	phase I/II	i.t. BO-112 (TLR3 agonist) with ablative radiotherapy and i.v. nivolumab (anti-PD-1)	2022
**NCT04796194**	Active	ORR, OS, CBR	NO	advanced melanoma	phase II	i.t. LTX-315 (oncolytic peptide, TLR7 activation) and i.v. pembrolizumab (anti-PD-1)	2021
**NCT04633278**	Completed	ORR	NO	metastatic head and neck squamous cell carcinoma	phase II	i.t. or s.c.CMP-001 (oligonucleotide, TLR9 activation) and i.v. pembrolizumab (anti-PD-1)	2021
**NCT04698187**	Completed	ORR	NO	unresectable and metastatic melanoma	phase II	i.t. CMP-001 (oligonucleotide, TLR9 activation) and i.v. nivolumab (anti-PD-1)	2021
**2020-003921-51**<br>**France**	Completed	ORR, CR, PR	NO	advanced melanoma	Phase II	i.t. BO-112 (TLR3 agonist) and i.v. pembrolizumab (anti-PD-1)	2020
**NCT04196283**	Completed	AE, changes in laboratory tests	NO	metastatic head and neck squamous cell carcinoma	phase I	i.t. tilsotolimod (TLR9 agonist) and i.v. ABBV-368 (anti-OX40), i.v. ABBV-181 (anti-PD-1), and i.v. nab-paclitaxel	2020
**NCT04270864**	Active. not recruiting	DLT	NO	advanced solid cancer	phase I	i.t. tilsotolimod (TLR9) and i.t. ipilimumab (anti-CTLA-4), and i.v. nivolumab (anti-PD-1)	2020

**NCT04147234**	Completed	MTD, DLT	YES (no DLT observed, MTD not achieved, stable disease in 53.3% of patients) (2)	advanced and metastatic solid tumors	phase I	i.t. BI 1387446 (STING agonist) and i.v. ezabenlimab (anti-PD-1)	2020
**NCT04570332**	Active, not recruiting	CR, PR	NO	unresectable melanoma	phase II	i.v. pembrolizumab (anti-PD-1) followed by i.t. BO-112 (TLR3 agonist)	2020
**NCT04220866**	Completed	ORR	YES (ORR 50%, PFS 6.4 months)	metastatic or unresectable head and neck cancer	phase II	i.t. ulevostinag (STING agonist) with i.v. pembrolizumab (anti-PD-1)	2020
**NCT04612530**	Recruiting	AE	NO	metastatic pancreatic cancer	phase I	i.v. FOLFIRINOX, followed by i.t. CpG (TLR9 agonist), electroporation, and i.v. nivolumab (anti-PD-1)	2020
**NCT04050085**	Completed	AE	NO	metastatic pancreatic cancer	phase I	i.t. SD-101 (oligonucleotide, TLR9 activation), radiotherapy, i.v. nivolumab (anti-PD-1)	2019
**NCT03865082**	Active, not recruiting	efficacy, response duration	NO	solid cancer	phase II	i.t. tilsotolimod (TLR9 agonist), i.v. Nivolumab (anti-PD-1) and Ipilimumab (anti-CTLA-4)	2019

**NCT03789097**	Recruiting	DLT	NO	metastatic breast cancer	phase I/II	radiotherapy, i.t. Flt3L and Poly ICLC, and i.v. Pembrolizumab	2019
**NCT03831295**	Completed	DLT	NO	advanced and metastatic solid cancer	phase I	i.t. SD-101 (TLR9 agonist) and i.t. and i.v. BMS 986178 (anti-OX40)	2019
**NCT04116320**	Active, not recruiting	DLT	NO	advanced solid cancer	phase I	focused ultrasound ablation, i.t. Hiltonol (TLR3 agonist), and anti-PD-1 (not specified)	2019
**NCT03956680**	Completed	DLT, AE, SAE, changes in laboratory tests, mortality	NO	advanced solid cancer	phase I	i.t. BMS-986301 (STING agonist) and i.v. nivolumab (anti-PD-1) and ipilimumab (anti-CTLA-4)	2019
**NCT03507699**	Completed	DLT	NO	metastatic colorectal cancer	phase I	i.t. CMP-001 (TLR9 agonist), i.v. nivolumab (anti-PD-1) and ipilimumab (anti-CTLA-4) with/without radiotherapy	2018
**NCT03007732**	Active, not recruiting	TEAE	NO	metastatic prostate cancer	phase II	i.t. SD-101 (oligonucleotide, TLR9 activation), i.m. leuprolide acetate (gonadotropin-releasing hormone agonist), i.v. pembrolizumab (anti-PD-1), p.o. prednisone (corticosteroid), and radiotherapy	2017

	**NCT03249792**	Completed	DLT, AE	NO	advanced and metastatic solid cancer	phase I	i.t. MK-2118 (STING agonist) and i.v. pembrolizumab (anti-PD-1)	2017
	**NCT03010176**	Completed	DLT, AE	YES (SAE Cut/Subcut Lesions 50%)	advanced and metastatic solid tumors	phase I	i.t. ulevostinag (STING agonist) and i.v. pembrolizumab (anti-PD-1)	2017
	**NCT02680184**	Completed	RP2D, TEAE	NO	advanced melanoma	phase I	i.t. CMP-001 (oligonucleotide, TLR9 activation) and i.v. pembrolizumab (anti-PD-1)	2016
	**NCT02668770**	Active, not recruiting	MTD	NO	unresectable and metastatic melanoma	phase I	i.t. MGN1703 (TLR9 agonist) and i.v. ipilimumab (anti-CTLA-4)	2016
	**NCT02644967**	Completed	ORR	YES<br>(CR 3.8%, PR 17%)	metastatic melanoma	phase II	i.t. IMO-2125 (TLR9 agonist) and i.v. Ipilimumab (anti-CTLA-4)	2015
Cytokines	**NCT06249048**	Recruiting	DLT, TEAE, SAE	NO	advanced solid tumors	phase I/II	i.t. STX-001 (IL-12 mRNA) and i.v. pembrolizumab (anti-PD-1)	2024
	**NCT06124001**	Not yet recruiting	MTD, DLT, AE, SAE, ORR	NO	advanced hepatocellular carcinoma	phase I/II	i.t. IL12/15-PDL1B (herpes simplex virus encoding IL-12, IL-15, IL-15RA) and i.v. camrelizumab (anti-PD-1)	2023

	**NCT06284590**	Active. not recruiting	ORR	NO	stage III-IV melanoma	phase II	i.t. L19TNF (antibody-cytokine protein) and i.v. pembrolizumab (anti-PD-1)	2023
	**NCT05162118**	Recruiting	MTD, DLT, AE, SAE, DCR, RP2D	NO	advanced pancreatic cancer	phase I/II	i.t. VG161 (herpes simplex virus expressing IL-12, IL-15, IL-15Ra and PD-L1 blocking peptide) and i.v. nivolumab (anti-PD-1)	2022
	**NCT06008925**	Recruiting	RP2D/MTD	NO	metastatic gastric cancer	phase I/II	i.t. VG161 (herpes simplex virus expressing IL-12, IL-15, IL-15. IL-15Rα, and a PD-1/PD-L1 blocking peptide) alone or in combination with i.v. nivolumab (anti-PD-1)	2022
	**NCT04725331**	Recruiting	phase I AE and RDPB, phase IIa iORR and iDCR	NO	metastatic or advanced solid tumors	phase I/II	i.t. BT-001 (vaccinia virus expressing 4-E03 and GM-CSF) alone or with i.v. pembrolizumab (anti-PD-1)	2021
	**NCT04526730**	Active, not recruiting	pCR	NO	advanced melanoma	phase II	i.t. tavokinogene telseplasmid (TAVO) (DNA plasmid encoding IL-12), electroporation, i.v. nivolumab (anti-PD-1) and surgical resection, followed by i.v. nivolumab (anti-PD-1)	2020
	**NCT03567720**	Active, not recruiting	ORR	NO	locally advanced and metastatic TNBC	phase II	i.t. TAVO, electroporation, i.v. pembrolizumab (anti-PD-1), i.v. nab-paclitaxel,	2018

							gemcitabine plus carboplatin	
	**NCT03132675**	Active, not recruiting	ORR	NO	unresectable and metastatic melanoma	phase II	i.t. TAVO, electroporation, and i.v. pembrolizumab (anti-PD-1)	2017
	**NCT03954067**	Completed	DLT, AE,	NO	advanced and metastatic solid cancer	phase I	i.t. ASP9801 (vaccinia virus encoding IL-7 and IL-12) and i.v. pembrolizumab (anti-PD-1)	2019
	**NCT04050436**	Active, not recruiting	ORR, CRR	NO	advanced squamous skin cancer	phase II	i.t. RP1 (herpes simplex virus encoding GM-CSF) and i.v. cemiplimab (anti-PD-1)	2019
	**NCT03636477**	Completed	safety and tolerability	NO	recurrent glioblastoma	phase I	i.t. Ad-RTS-hIL-12 (adenoviral vector encoding IL-12), p.o. veledimex (small molecule), and i.v. nivolumab (anti-PD-1)	2018
	**NCT03294083**	Active, not recruiting	MTD, MFD, ORR, AE	NO	metastatic renal cell carcinoma	phase I/II	i.t. JX-594 (vaccinia virus expressing GM-CSF) and i.v. cemiplimab (anti-PD-1)	2018
	**NCT03003676**	Completed	TEAE (ORR for 3 doses of ONCOS-102 37.5%, SAE 44.44%)	YES	advanced melanoma	phase I	i.v. cyclophosphamide, followed by i.t. ONCOS-102 (adenovirus encoding GM-CSF) and i.v. pembrolizumab (anti-PD-1)	2016

**NCT02493361**	Completed	ORR	YES (AE 12.5%)	metastatic melanoma	phase II	i.t. pIL-12 in combination with i.v. pembrolizumab (anti-PD-1)	2015
**NCT02423902**	Completed	SAE, AE	NO	metastatic breast cancer	phase I/II	i.t. Ad-RTS-hIL-12 (adenoviral vector expressing IL-12) and p.o. veledimex (small molecule)	2015
**NCT01672450**	Completed	AE	NO	unresectable melanoma	phase I	i.t. IL-2 and i.t. ipilimumab (anti-CTLA-4)	2012
**NCT01480323**	Completed	Control rate	NO	stage IV melanoma	phase II	i.t. interleukin-2 and i.v. ipilimumab (anti-CTLA-4)	2012

Abbreviations: DLS, dose-limiting toxicities; ORR, overall response rate; MTD, maximum tolerated dose; OS, overall survival; PFS, progression-free survival; RP2D, recommended phase II dose; DCR, disease control rate; AE, adverse events; SAE, serious adverse events; RDPB, the recommended dose for part B; iORR, immune overall response rate; iDCR, immune disease control rate; DLT, dose-limiting toxicity; TEAE, treatment-emergent adverse events; CBR, clinical benefit rate; mPR,; BORR, Best overall response rate; RLT, regimen limiting toxicity; RP2D, recommended phase II dose; pCR, pathologic complete response rate; CBR, clinical benefit rate; TNBC, triple-negative breast cancer; i.d., intradermal. Data were taken from www.clinicaltrials.gov to November 18th, 2024. Terminated, withdrawn, and unknown-status clinical trials were excluded.

Supplementary Table 2| **Timing of FDA-approved and clinical trial therapy combinations for the treatment of locally advanced and metastatic solid cancers.**

	Drug Name	Condition	Combination	Treatment Timing
**Approved Systemic Therapies for the Treatment of Locally Advanced and Metastatic Cancers**				
Checkpoint Inhibitor	i.v. pembrolizumab (anti-PD1)	metastatic or unresectable HNSCC	i.v. platinum and i.v. fluorouracil	pembrolizumab every 3 or 6 weeks; before chemotherapy when administered on the same day
		locally advanced or metastatic UC	i.v. enfortumab vedotin (anti-nectin-4 antibody-microtubule inhibitor conjugate)	pembrolizumab every 3 weeks or 6 weeks. After enfortunab when administered on the same day
		locally advanced or metastatic BTC	i.v. gemcitabine and i.v. cisplatin	pembrolizumab every 3 or 6 weeks; before chemotherapy when administered on the same day
		unresectable or metastatic TNBC	i.v. chemotherapy	pembrolizumab every 3 or 6 weeks; before chemotherapy when administered on the same day
	i.v. atezolizumab (anti-PD-L1)	metastatic HCC	i.v. bevacizumab (anti-VEGF)	atezolizumab every 2, 3, or 4 weeks; before bevacizumab when administered on the same day
		unresectable or metastatic melanoma	p.o. cobimetinib and p.o. vemurafenib (kinase inhibitors)	cobimetinib (21 days on and 7 days off) before atezolizumab (every 2, 3, or 4 weeks based on concentration), and vemurafenib (1-28 days)
	i.v. ipilimumab (anti-CTLA-4)	unresectable or metastatic melanoma	i.v. nivolumab (anti-PD-1)	simultaneously
Targeted Therapy	i.v. trastuzumab (anti-HER2) and hyaluronidase	HER2-positive metastatic breast cancer	i.v. trastuzumab and i.v. docetaxel	trastuzumab and hyaluronidase and trastuzumab in any order; taxane after

				trastuzumab and hyaluronidase and trastuzumab
	i.v. ramicurimab (anti-VEGFR)	advanced or metastatic, gastric or GEJ	paclitaxel (i.v.)	ramicurimab every 2 weeks; before paclitaxel when administered on the same day
		metastatic CRC	i.v. FOLFIRI (irinotecan, folinic acid, and fluorouracil)	ramicurimab every 2 weeks; before FOLFIRI when administered on the same day
	i.v. cetuximab (anti-EGFR)	locally advanced SCCHN	radiotherapy	cetuximab one week before radiotherapy; cetuximab every week (6–7 weeks); cetuximab administration 1 hour before radiotherapy
		metastatic CRC	i.v. FOLFIRI (irinotecan, fluorouracil, leucovorin	cetuximab weekly or biweekly; cetuximab administration 1 hour before FOLFIRI
	i.v. naxitamab (anti-GD2)	refractory high-risk neuroblastoma	s.c. GM-CSF	naxitamab after GM-CSF (day 1, 3, 5); cycles repeated every 4 weeks, followed by 5 additional cycles
**Intratumoral Clinical Trials for the Treatment of Locally Advanced and Metastatic Cancers**				
Checkpoint Inhibitor	**NCT04270864**<br>i.t. ipilimumab (anti-CTLA-4)	advanced cancer	i.v. nivolumab (anti-PD-1) and i.t. tilsotolimod (TLR9 agonist)	once every 3 weeks
	**NCT03982121**<br>i.t. ipilimumab (anti-CTLA-4)	colorectal liver metastases	i.t. GLA-SE (TLR4 agonist), i.v. FOLFOX (leucovorin calcium, fluorouracil, and oxaliplatin), and i.v. nivolumab (anti-PD-1)	Not specified

	**NCT01672450**<br>i.t. ipilimumab<br>(anti-CTLA-4)	stage III-IV melanoma	i.t. IL-2	IL-2 on days 1, 3, and 5 for 2 weeks, then on days 1 and 4 for 6 weeks; ipilimumab once a week for 8 weeks
Cell Therapy	**NCT02432846**<br>i.t. intuvax (DCs)	metastatic renal cell carcinoma	i.v. sunitinib (kinase inhibitor)	2 intuvax injections 14 days apart, followed by nephrectomy and sunitinib
	**NCT03747744**<br>i.t. autologous DCs	subcutaneous/cutaneous, soft tissue metastases	i.t. T-VEC	Not specified
Immunomodulator	**NCT02493361**<br>i.t. pIL-12	metastatic melanoma	i.v. pembrolizumab (anti-PD-1)	pIL-12 on days 1,5,8 of odd cycles; pembrolizumab on days 1 of each cycle
	**NCT04220866**<br>Ulevostinag (STING agonist)	metastatic and unresectable head and neck squamous cell carcinoma	i.v. pembrolizumab (anti-PD-1)	ulevostinag on day 1 of cycles 1-35; pembrolizumab on day 1 for up to 35 cycles
	**NCT03507699**<br>i.t. CMP-001 (TLR9 agonist)	metastatic colorectal cancer	s.c. CMP-001 (TLR9 agonist), i.v. nivolumab (anti-PD-1) and ipilimumab (anti-CTLA-4) with/without radiotherapy	Three radiation treatments for liver metastasis, nivolumab every 2 weeks, ipilimumab every 6 weeks, and CMP-001 injected once in the liver metastases and four times s.c. for up to 6 weeks
	**NCT01480323**<br>i.t. IL-2	stage IV melanoma	i.v. ipilimumab (anti-CTLA-4)	IL-2 on days 1, 4, 8, 11, 15, 18, 22, and 25; ipilimumab on days 2, 23, 44, 65
	**NCT04370587**<br>i.t. T3011 (oncolytic virus)	advanced and metastatic solid tumors	i.v. pembrolizumab (anti-PD-1)	T3011 and pembrolizumab once every 3 weeks

	**NCT02307149**<br><br>i.t. CAVATAK (Coxsackievirus)	advanced melanoma	i.v. ipilimumab (anti-CTLA-4)	Not specified
	**NCT03990493**<br><br>i.t. PV-001-DV (Dengue virus)	advanced melanoma	i.v. autologous DCs	PV-001-DV once; autologous DCs every 3 weeks for up to 4 treatments
Monoclonal Antibody	**NCT06205849**<br><br>i.t. anti-CD40	locally advanced pancreatic cancer	irreversible electroporation	Not specified
	**NCT04547777**<br><br>i.t. 2141-V11 (anti-CD40)	stage III-IV glioma	i.t. D2C7 (antibody-drug conjugate)	D2C7 for 72 hours, followed by 2141-V11 infusion for up to 7 hours

Abbreviations: HNSCC, head and neck squamous cell carcinoma; UC, urothelial cancer; BTC, biliary tract cancer; TNBC, triple-negative breast cancer; HCC, hepatocellular carcinoma; GEJ, gastroesophageal junction adenocarcinoma; CRC, colorectal cancer; SCCHN, squamous cell carcinoma of head and neck; GM-CSF, granulocyte-macrophage colony-stimulating factor; i.v., intravenous; s.c., subcutaneous; p.o., per os. Data were taken from www.dailymed.nlm.nih.go and www.clinicaltrials.gov to July 18th, 2024.

References

1. Nassiri F, Patil V, Yefet LS, Singh O, Liu J, Dang RMA, et al. Oncolytic DNX-2401 virotherapy plus pembrolizumab in recurrent glioblastoma: a phase 1/2 trial. Nature Medicine. 2023;29(6):1370-8. http://dx.doi.org/10.1038/s41591-023-02347-y.

2. Calvo E, Garralda E, Alonso G, Gambardella V, Parkes EE, Thompson J, et al. 1030P Phase I, first-in-human trial evaluating the STING agonist BI 1387446 alone and in combination with ezabenlimab in solid tumors. Annals of Oncology. 2023;34:S626. http://dx.doi.org/10.1016/j.annonc.2023.09.2169.
